# Probabilistic liver atlas construction

**DOI:** 10.1186/s12938-016-0305-8

**Published:** 2017-01-13

**Authors:** Esther Dura, Juan Domingo, Guillermo Ayala, Luis Marti-Bonmati, E. Goceri

**Affiliations:** 1 Department of Informatics, School of Engineering, University of Valencia, Avda. de la Universidad, 46100 Burjasot, Spain; 2 Department of Statistics and Operations Research, University of Valencia, Avda. Vicent Andrés Estellés, 1, 46100 Burjasot, Spain; 3 Universitary and Politechnic Hospital La Fe, Valencia, Spain; 4 Department of Computer Engineering, Akdeniz University, Antalya, Turkey

**Keywords:** Anatomical atlas, Probabilistic atlas, Generalized linear model, Coregistration method, Atlas variability

## Abstract

**Background:**

Anatomical atlases are 3D volumes or shapes representing an organ or structure of the human body. They contain either the prototypical shape of the object of interest together with other shapes representing its statistical variations (statistical atlas) or a probability map of belonging to the object (probabilistic atlas). Probabilistic atlases are mostly built with simple estimations only involving the data at each spatial location.

**Results:**

A new method for probabilistic atlas construction that uses a generalized linear model is proposed. This method aims to improve the estimation of the probability to be covered by the liver. Furthermore, all methods to build an atlas involve previous coregistration of the sample of shapes available. The influence of the geometrical transformation adopted for registration in the quality of the final atlas has not been sufficiently investigated. The ability of an atlas to adapt to a new case is one of the most important quality criteria that should be taken into account. The presented experiments show that some methods for atlas construction are severely affected by the previous coregistration step.

**Conclusion:**

We show the good performance of the new approach. Furthermore, results suggest that extremely flexible registration methods are not always beneficial, since they can reduce the variability of the atlas and hence its ability to give sensible values of probability when used as an aid in segmentation of new cases.

## Background

With the increasing capabilities of computers, computational anatomy has become an area of interest for medical diagnosis. In particular, lesion detection and surgery planning are very demanding tasks that require extracting the volume of an organ first. For this purpose, image analysis algorithms such as registration or segmentation have to be applied. However without prior knowledge of the organ, in terms of spatial and shape information as well as variability, is difficult to provide a reliable volume. To overcome this limitation most of the current state-of-the-art image analysis techniques use anatomical atlases as a form of priori information like [1–3]. Atlases provide a map or chart of the anatomy; they usually have geometric information about curve points or surfaces or label information about voxels which correspond to anatomical regions or functions. The atlases are normally constructed from populations of subjects and can be classified in two main categories: statistical and probabilistic atlases. Both types were developed with different purposes and so, the techniques for their construction are quite different, too. This paper focuses mainly on the comparison of probabilistic atlases, although a simple example of statistical atlas is also tried, so the next sections of this introduction will make reference to both types.

### Statistical atlases

Statistical atlases provide a set of binary 3D shapes representing the prototypical shape (mean) and different modes of variation. This allows the analysis of the shape variability, as they capture the organ variability within justified bounds. The most common approach to analyze the shape variability is based on statistical shape models [4]. They have been used for recognition and segmentation of different human organs and even more specifically, for the description, analysis and modeling of the human liver. Constructing statistical shape models consists of extracting the mean shape and certain number of modes of variation from a collection of training samples. The modes of variation that best describe the shape of the liver, are usually accomplished by using Principal Component Analysis (PCA, [5]) as in [6, 7] or [8] in which pattern recognition techniques are used for classification of liver cirrhosis. Other interesting applications include the development of occupant finite element dummies used in crash biomechanics (see [9], in which the authors use an Iterated Closest Point method for alignment followed by Principal Component Analysis (PCA) or [10] which is a more evolved approach that starts with automatically placed landmarks).

Statistical atlas based methods have been applied for segmentation of organs such as the liver or the pancreas, see [11] and [12]. Nevertheless it is difficult to build a mean shape of an organ given the large inter-subject differences or irregularities of the shapes, therefore the mean shape of a particular set may be biased. Besides, when the eigenvalues of the PCA matrix, or at least two of them are very similar, it is difficult to have a good representation. An appropriate way to cope with that is to use spherical harmonics, as in [6]. Another of the main drawbacks of statistical atlases is that they only provide possibles modes of variation (which are useful for identification of structures, quantitative analysis and registration) but are more difficult to be used in segmentation. A notable exception that successfully uses statistical shape models to segment the liver in difficult cases is [13]; the key there consists of using not a single model for all the shape but a multi-level local region based model.

### Probabilistic atlases

In the framework of computational anatomy, a probabilistic atlas it is not only the average boundary of an organ or structure of the body, but the confidence or the complete spatial distribution of probabilities that a voxel belongs to an organ. Interesting examples can be found in [2, 14–16] or [17]. For each voxel the probability is computed from the frequency of occurrence of mapped, mostly manual, segmentations. The main advantage of using probabilistic atlases regarding statistical atlases is that they can be very useful to find the most probable edges of a patient’s organ or structure of the body, specially when the signal-to-noise level of the images is low.

In this paper we are particularly interested in probabilistic atlases for their further use in segmentation. Some segmentation algorithms can interpret the value given by the atlas as an a-priori probability and use Bayesian methods to update it using the values of the signal at that voxel or at neighbouring voxels as new information like in [18]). Other algorithms can interpret it as a possibility of belonging to the set of voxels that are the relevant structure and rely on fuzzy techniques and finally others can use the value as initial function to apply level-set techniques. In the rest of this section it will explained how probabilistic atlases have been built together with the improvement applied to get a more accurate result.

So far one of the main approaches used for the construction of probabilistic atlases is to register the binary shapes in the sample and look at each voxel to see how many of the shapes cover it. This, divided by the number of shapes in the sample, is a crude measure of the probability that voxel has of belonging to the ideal shape. This is used for example in [2]. We will formalize this as the coverage function. Nevertheless, it is worth noting that there are other more sophisticated approaches that estimate the likelihood function for each class (such in the case of multi-organ atlases) using a Gaussian kernel and that use not only one, but several atlases to compose the final one according to the case (patient) for which the atlas is to be used (see [19]).

Other possibilities for building probabilistic atlases use the distance function and transformations of it. Intuitively, the distance function associated with a binary shape is a function from the 3D space to the real numbers and measures how far each point is from the shape. There are two variants: unsigned distance function, for which all the points of the shape get a null value and those outside get the distance to the closest point of the shape, and signed distance for which the outer points get a value as before and the inner points get the value of their distance to the closest border with opposite (negative) sign. There are few approaches that use the distance function to build atlases; relevant works from Pohl are [20] and [21]. In this work signed distance function is related to the logarithm of odds ratio (LogOdds). The LogOdds encode the certainty of objects’ boundaries in images. Unlike this work, the main idea presented in this paper regarding atlas construction is the combination of both approaches, the coverage function and the distance function, using a generalized linear model (GLM). This will be further explained in “Probabilistic atlas: mathematical formulation” section.

### Influence of the registration

It is important to note that for both approaches, statistical and probabilistic, the initial raw data are examples of correctly segmented binary shapes. The procedure for segmentation is of crucial importance to obtain a good atlas. Another extremely important point is the alignment (in this context, coregistration) of the binary shapes of the sample, which is the first step before building the atlas. Hence one of the main goals of this work is to investigate the influence of the alignment for the construction of atlas. Several models for registration can be chosen, from the simplest ones (rigid transformations composed by a translation plus a rotation) to the more complex and flexible models like local deformations. The most complex models allow an almost perfect registration but they contain many parameters that must be optimized. Moreover, the use of very flexible models in this context is not always recommended, since by adjusting perfectly the shapes to one of them (or to a prototypical shape) the intrinsic variability is lost and the atlas does not properly reflect an ideal shape but fits perfectly only in one of the sample shapes.

The possible models of geometric transformation used for building the atlas will be studied in more detail in “Different models for registration” section.

### Contributions

The main contributions of this paper are twofold. In “Probabilistic atlas: mathematical formulation” section, we propose a new method for probabilistic atlas construction that integrates in a coherent way the two most used techniques: those based on the coverage function and those based on the distance function. Later in “Numerical results” section, a systematic study is done with a real case (liver atlas construction) to assess the influence of the method used for atlas construction and of the previous step of coregistration. Even applied to the particular case of an organ (liver), the evaluation method can be reproduced for any other organ or shape.

## Anatomical atlas building

In general, all kinds of atlases require a previous segmentation of the organ or structure of interest since inputs are binary shapes. In our case, the liver to which the method is applied was manually segmented by an expert radiologist using magnetic resonance images (MRI explorations) from 19 subjects. Details on the sample composition and acquisition methods and on the digital and real dimensions of the acquired images will be explained in “Experiment condition and materials” section.

Segmentation can be performed either by automatic or manual methods, or by a combination of both. Examples of the first approach are scarce in the case of the liver, due to the great difficulty of its segmentation [22]. An organ very hard to segment, too, is the brain (see [23]). Obviously, an inaccurate segmentation will diminish the quality of the atlas, specially in the limiting surface of the organ, but it is extremely difficult, if not impossible, to evaluate the importance of this factor since we have no access to a sufficiently large database of the same organ present in the same cases and segmented with different methods. This is why we have not attempted the quantification. Nevertheless, should these examples be available, the evaluation method could be applied, too, without changes.

It is important to note that one of the main purposes of an atlas is to use it for segmentation. This means that the atlas must capture to some extent the mean shape, but also the variations so it can reasonably adapt to a new case not previously seen and of course not used for the construction of the atlas. This aspect is treated with special care in this work.

Once the structures has been segmented and binary 3D shapes of the organ of interest are available, they must be aligned (in the language of medical image, registered). The registration process involves several choices. First, the different instances can be aligned with one of the cases but then, it must be chosen with which one. Otherwise, if the instances are registered to a common reference frame, such frame must be chosen, too. The next step is the selection of a model of geometric transformation. The most popular ones, in order of complexity, are translations (scarcely used for these purposes), rigid transformations (translation plus rotation), rigid transformations with further global scaling, global affine deformations and locally variant deformations [24]. The more complex ones give more accurate and visually pleasant results but, as we will argue later, this not always leads to a better atlas, since it reduces the variability, and therefore the ability of an atlas to capture the shape variations and be useful for segmenting a new case. The parameters of the geometric transformations have to be chosen so as to minimize a chosen similarity function. The choice of this function may be also a relevant factor but we considered its analysis out of the scope of this paper; therefore in our experiments we have considered the same function for all geometric models: the function to be minimized is the mean squared intensity difference. The different models compared in this paper will be fully described in “Different models for registration” section.

### Different models for registration

As stated in “Anatomical atlas building” section, the influence of the geometric model used for coregistration before the actual construction of the atlas cannot be underestimated. Few studies have been conducted to evaluate this point, but some of them are very exhaustive like [25] for computed tomography (CT) images of organs in the abdominal cavity. Most of the investigated strategies are local and non linear deformations; up to 12 of them are compared in [26]. We are also interested in comparing very simple registration models and, as not every possible model can be tested, the decision has been to use at least one significant representative of each transformation type, from the simplest to the more complex ones. They include a rigid transformation (translation plus rotation), a global scaling (translation plus rotation plus scaling about the center), an affine global model and a local deformable model. As it will be shown in “Numerical results” section, the quality of fit of these transformations with respect to the individuals used to estimate them increase with the complexity, as it can be expected, up to the point in which the coregistered shapes coincide in a virtually indistinguishable way (for the local deformable model). Nevertheless, what seems an advantage becomes an inconvenience when the resulting atlas is compared with a new shape (not used for constructing it). The main objective of this paper is precisely to show how to balance the complexity so that the result is accurate enough but also keeps sufficient shape variability.

The used geometric transformations (see [27], chapter 9) are as follows:

- Rigid transformation consists of translation of all the shapes to a common origin of their reference frames (3 free parameters in ${\mathbb R}^3$) plus rotations about that point (another 3 free angles). This is expressed as
  $$\begin{aligned} \left( \begin{array}{c} x'\\ y'\\ z'\\ \end{array}\right) = \left( \begin{array}{ccc} \cos \phi &{} -\sin \phi &{} 0\\ \sin \phi &{} \cos \phi &{} 0\\ 0 &{} 0 &{} 1\\ \end{array} \right) \left( \begin{array}{ccc} \cos \varphi &{} 0 &{} \sin \varphi \\ 0 &{} 1 &{} 0\\ -\sin \varphi &{} 0 &{} \cos \varphi \\ \end{array} \right) \left( \begin{array}{ccc} 1 &{} 0 &{} 0\\ 0 &{} \cos \psi &{} -\sin \psi \\ 0 &{} \sin \psi &{} \cos \psi \\ \end{array} \right) \left( \begin{array}{c} x\\ y\\ z\\ \end{array} \right) + \left( \begin{array}{c} x_c\\ y_c\\ z_c\\ \end{array} \right) \end{aligned}$$
  being $(x',y',z')$ the coordinates of the transformed point, (*x*, *y*, *z*) those of the original point, $(x_c,y_c,z_c)$ the location of the translated origin and $(\phi ,\varphi ,\psi )$ the three rotation angles about each axis (other choices of angular representation can be used, too). The usual way of determining the transformation is to search for the minimum of a goodness of fit function (optimize) on the six free parameters. For this and all the other transformations used the evaluation function to be minimized was the sum of squared differences between the destination and the transformed original image.
- Global rescaling consists of the same rigid transformation formerly described plus a rescaling of the shape (homothety) about the center of common alignment. This gives a total of 7 parameters. Analytic expression is
  $$\begin{aligned} \left( \begin{array}{c} x'\\ y'\\ z'\\ \end{array}\right) = K_h\left( \begin{array}{ccc} \cos \phi &{} -\sin \phi &{} 0\\ \sin \phi &{} \cos \phi &{} 0\\ 0 &{} 0 &{} 1 \end{array} \right) \left( \begin{array}{ccc} \cos \varphi &{} 0 &{} \sin \varphi \\ 0 &{} 1 &{} 0\\ -\sin \varphi &{} 0 &{} \cos \varphi \\ \end{array} \right) \left( \begin{array}{ccc} 1 &{} 0 &{} 0\\ 0 &{} \cos \psi &{} -\sin \psi \\ 0 &{} \sin \psi &{} \cos \psi \\ \end{array} \right) \left( \begin{array}{c} x\\ y\\ z\\ \end{array} \right) + \left( \begin{array}{c} x_c\\ y_c\\ z_c\\ \end{array} \right) \end{aligned}$$
  where the meaning of the symbols is as before, with the only addition of $K_h$ (the homothety constant). Determination of the value of the parameters this time was done in batches of two successive optimization steps: rigid transformation (6 parameters) and homothety constant.
- Affine global model applies a linear transformation of the original coordinates with an unrestricted $3\times 3$ matrix plus an unrestricted displacement, which adds up to 12 parameters. Equation is
  $$\begin{aligned} \left( \begin{array}{c} x'\\ y'\\ z'\\ \end{array}\right) = \left( \begin{array}{ccc} m_{00} &{} m_{01} &{} m_{02}\\ m_{10} &{} m_{11} &{} m_{12}\\ m_{20} &{} m_{21} &{} m_{22}\\ \end{array}\right) \left( \begin{array}{c} x\\ y\\ z\\ \end{array} \right) + \left( \begin{array}{c} x_c\\ y_c\\ z_c\\ \end{array} \right) \end{aligned}$$
  where all $m_{ij}$ and $(x_c,y_c,z_c)$ keep constant values [by global transformation we mean they do not depend on the (*x*, *y*, *z*) point]. The 12 parameters of this transformation are determined by minimization as before.
- As a representative example of a local deformation algorithm, a slight variation of the original demons algorithm from Thirion [28] has been chosen. As stated in the original paper, “This method considers the object boundaries in one image as semi-permeable membranes and lets the other image, considered as a deformable grid model, diffuse through these interfaces, by the action of effectors situated between the membranes”. The result, along with the deformed image, is a vector field (the deformation field) that describes the displacement of each point.

All these methods need the choice of a reference case to which all others are registered. It is not clear in real situations which case to adopt as reference. To overcome this difficulty we have systematically registered to each of our available cases and we have chosen as reference that which leads to a smaller average value of the evaluation function. This is to get the optimal result for each method.

### Probabilistic atlas: mathematical formulation

Once the shapes in the sample have been segmented and coregistered the probabilistic atlas can be finally built. As previously stated, the most widely used way of building probabilistic atlases consists of aligning the shapes and seeing then how many shapes cover each voxel. The formalization uses random set theory [29]. A random (3D) random set is a probabilistic model whose realizations are random compact subsets of the 3D Euclidean space. From now on, we will work with random compact sets denoted as $\Phi$. The realizations of a random compact set are binary shapes: sets of points, in this case of ${\mathbb R}^3$ (but in general, ${\mathbb R}^d$) with the only restriction of being compact (but not necessarily convex). For a fixed shape *S*, and for any point $x\in {\mathbb R}^d$, $1_S(x)$ will stand for the set indicator function, i.e.: $1_S(x)= 1$ if $x \in S$ and 0 otherwise. For a random compact set $\Phi$ the value $1_{\Phi }(x)$ is a random variable taking values in $\left\{ 0,1\right\}$. The probability of a point *x* belongs to the random set $\Phi$ will be denoted by *p*(*x*) i.e. $p(x) = E \left( 1_{\Phi }(x) \right) = P(x \in \Phi )$. If we have a random sample of $\Phi$ i.e. independent and identically distributed (as $\Phi$) random compact sets $\Phi _1, \ldots , \Phi _n$, the (natural) unbiased estimator of *p*(*x*) would be

1 $$\begin{aligned} \hat{p}_1(x) = \sum _{i=1}^n 1_{\Phi _i}(x). \end{aligned}$$

which counts the number of shapes in the sample to which point *x* belongs. Given the realizations, $\phi _1, \ldots , \phi _n$, the corresponding estimate (denoted as the estimator) would be $\hat{p}_1(x) = \sum _{i=1}^n 1_{\phi _i}(x)$. This definition (and the mentioned estimation method) for *p*(*x*) is interpreted as the probabilistic atlas in most references [2, 14, 16, 30] and from now on we will refer to it as normalized coverage function (NCF).

Certainly, *p*(*x*) captures in a very intuitive way the classical notion of probability. Also, its threshold below 0.5 is sometimes considered as mean shape, and indeed it is a particular case of the so-called V’orobev mean [31]. But this definition for *p*(*x*) has some drawbacks mainly related to the need to estimate the probability at each point in isolation, i.e.: considering the random variable giving the coverage at that point as independent of those of all other points. This makes the thresholds below a given value of *p*(*x*) (which are binary shapes) rougher than it would be expected of a summary shape.

A feasible alternative to solve this problem consists of using the distance function; concretely, on finding a sensible relationship between the probability and the value of the distance function at a given point or at some related points. The formal definitions are as follows: given a binary shape, *S*, $d_S(x)$ will be the signed distance function to *S*:

2 $$\begin{aligned} d_S(x)=\left\{ \begin{array}{ll} \min _{y\in \partial S}d(x,y) &{} \text{ if } x\notin S\\ 0 &{} { \text{ if } x \in \partial S}\\ -\min _{y\in \partial S}d(x,y) &{} \text{ if } x\in int(S), \end{array} \right. \end{aligned}$$

being *d*(*x*, *y*) the Euclidean distance between points *x* and *y*, $\partial S$ the boundary of *S* and *int*(*S*) the interior of the set *S*. In a similar way, $d_{\Phi }$ can be defined not for a fixed set but for a random set $\Phi$. In this case, $d_{\Phi }(x)$ is a random variable. Since $1_{\Phi }(x) = 0 \iff d(x) > 0$ and $1_{\Phi }(x) = 1 \iff d(x) \le 0$, it is clear that, given *d*(*x*), $1_{\Phi }(x)$ is known. Let the mean distance function $d_{\Phi }^*(x)$ be defined as

3 $$\begin{aligned} d_{\Phi }^*(x) = E \left( d(x,\Phi ) \right) . \end{aligned}$$

In practice, the mean distance function is estimated for a collection of samples $\phi _1,\ldots ,\phi _n$ as

4 $$\begin{aligned} \hat{d}_{\Phi }^*(x)=\sum _{i=1}^n \frac{d_{\phi _i}(x)}{n} \end{aligned}$$

Like $d_{\Phi }(x)$, $d_{\Phi }^*(x)$ is a signed quantity and similarly to the mean coverage, its threshold below some value gives a binary shape that can also be considered as a mean shape, being this time 0 a sensible threshold (a definition derived form the so-called Baddeley–Molchanov mean, [32]). The mean distance function is smooth so its thresholds are smoother than those of the mean coverage function. This is one of the reasons why we will attempt to estimate the function *p*(*x*) using information about the mean distance function. From now on, $d_{\Phi }^*(x)$ will be denoted simply as $d^*(x)$.

Assuming that $p(x)=f(d^*(x))$ (i.e.: the probability is a function of the mean distance function) the goal is to find a sensible link between both. But *p*(*x*) is in [0, 1] and $d^*(x)$ can take positive and negative values. We will resort to the usual approach in generalized linear models: link input and output through a cumulative distribution function, a non-decreasing function $F: {\mathbb R} \longrightarrow \left[ 0,1\right]$. From the value $d^*(x)$ its transformations using a set of basis functions denoted as $\varvec{v}(x) = (1,v_1(d^*(x)),\ldots ,v_{p-1}(d^*(x)))'$ will be considered ($\varvec{t'}$ denotes the transpose of $\varvec{t}$). The following model will be assumed:

$$\begin{aligned} p(x) = F(\beta \varvec{v}(x)) \end{aligned}$$

with $\varvec{\beta } = (\beta _0,\beta _1,\ldots ,\beta _{p-1})'$. The two usual choices for the link function *F* are the cumulative distribution functions corresponding either to the logistic distribution or the standard (Gaussian) distribution. In particular, we will use the logistic distribution so $p(x) = \frac{e^{\varvec{ \beta '} \varvec{v}(x)}}{1 + e^{\varvec{\beta } \varvec{v}(x)}}$. Let us consider a given point $x_0$. If *p*(*x*) is a smooth function then a constant value for *p*(*x*) in a ball centered at *x*, $B(x_0,h)$ with $h >0$, can be assumed. If $(x_j,1_{\phi _i}(x_j))$ with $j = 1,\ldots , J$ denote the points located within $B(x_0,h)$, being *J* the number of such points, the local pseudolikelihood function for the *i*-th realization $\phi _i$ is given by

5 $$\begin{aligned} \prod _{j=1}^J w(x_j,x_0) p(x_j)^{1_{\phi _i}(x_j)} (1-p(x_j))^{1- 1_{\phi _i}(x_j)}, \end{aligned}$$

where $w(x,x_0) = K(\parallel x- x_0\parallel /h)$ being *K* a kernel function and *h* a positive parameter, the bandwidth. But we have a random sample of $\Phi$ so the whole logarithm of likelihood (*loglikelihood*) function can be written as:

6 $$\begin{aligned} l(\beta ) &= \log L(\beta ) = \sum _{i=1}^n \sum _{j=1}^J \bigg ( \log (w(x_j,x_0)) \nonumber \\&\quad+ 1_{\Phi _i}(x_j) \log (p(x_j)) + (1- 1_{\Phi _i}(x_j)) \log (1-p(x_j)) \bigg ). \end{aligned}$$

Let $\hat{\beta }(x_0)$ be the vector of parameters that maximize this global likelihood, i.e.: $\hat{\beta }(x_0) = argmax_{\beta } \ l(\beta )$. $\hat{\beta }$ will be found using appropriate optimization methods. The estimator proposed for the probability function *p*(*x*) is finally:

7 $$\begin{aligned} \hat{p}(x_0) = \frac{e^{\hat{\varvec{\beta }}(x_0) \varvec{v}(x_0)}}{1 + e^{\hat{\varvec{\beta }}(x_0) \varvec{v}(x_0)}}. \end{aligned}$$

whose value at each location $x_0$ constitutes our atlas. From now on we will refer to it as generalized linear model (GLM).

Again, the thresholds of this new *p*(*x*) are also summary shapes (being the threshold at 0.5 analogous to the mean shape) but they are smoother and (as it will be shown in “Experimental study” section) more adjusted to new cases than the former definition based only on the coverage. The reason for the smoothness is twofold: on one hand, the distance function is by itself smoother than the coverage function and moreover, the linear model takes into account global information, not only the value at point *x*, to estimate *p*(*x*).

Finally, even centered on probabilistic atlases, the experiments of this paper make a comparison not only between the previous normalized coverage-based function (NCF) approach and our proposed generalized linear model (GLM) but also include a simple statistical atlas because this kind of atlas is commonly used so comparisons add a wider perspective. The shape taken as basis for comparison is taken from a set of landmarks automatically extracted from the samples. This brings some adjustments to have a fair comparison: first, being the result a binary shape and not a probability map, not all measures of goodness are appropriate. As stated in “Measures of goodness” section, the Dice dissimilarity, Jaccard distance and Hausdorff distance can be applied but not the mean probability. Another difficulty is that in our case input sample is composed by binary shapes, not by landmarks with whose spatial coordinates a model can be built. This has been solved by generating a network of landmarks fairly distributed on the surface of each shape and recreating a binary shape from the result (a set of points) by filling them using a viscous reconstruction. See details on its construction in “Implementation” section.

### Implementation

All the methods for atlas construction we analyze are formulated for the continuous space (${\mathbb R}^3$) but they have to be implemented using segmented discrete binary shapes.

In practical terms we really use the shape representation in digital form, i.e., as sets of pixels/voxels in the sampled space ${\mathbb Z}^3$ with a binary value assigned, 1 meaning that the point belongs to the shape and 0 that it doesn’t. As stated before, the available shapes have to be obtained either by segmenting real images to extract the pixels/voxels that fulfill a property, normally related to local visual features of the image like gray level, color, texture or similar or, as in our case, by manual segmentation by a radiologist. Coregistration with all the geometric transformation was implemented as C++ programs using the ITK toolbox [33]. The coverage functions and signed distance functions were obtained from the binary coregistered images, also with C++ programs using the ITK, as long as the final estimation of the probabilistic atlas. The optimization step in the GLM method required a call to a function included in the *locfit* package [34] of the statistical software R done from inside the C++ program using the *Rcpp* and *RInside* packages [35].

The linear model has been set up to use the simplest possible predictor (i.e.: $\varvec{v}(x) = (1,\hat{d}_{\Phi }^*(x))'$) and the kernel function *K* and bandwidth *h* used in the weight $w(x_j,x_0)$ of equation 5 have been left to the default values provided by the *locfit* package. The kernel function used is the tricube function given by $K(x) = \frac{70}{81}(1-|x|^3)^3$ for $-1 \le x \le 1$. The bandwidth selection has been used the default method given en [34]. See for details chapter 11 in [36]. A selected set of experiments were done using more complex predictors like a second-order model ($\varvec{v}(x) = (1,\hat{d}_{\Phi }^*(x), (\hat{d}_{\Phi }^*)^2(x))'$) with no significant differences in the final results. Illustrations of 3D results showing the atlas obtained by the NCF and GLM methods are shown in “Graphical illustration” section.

**Fig. 1:**
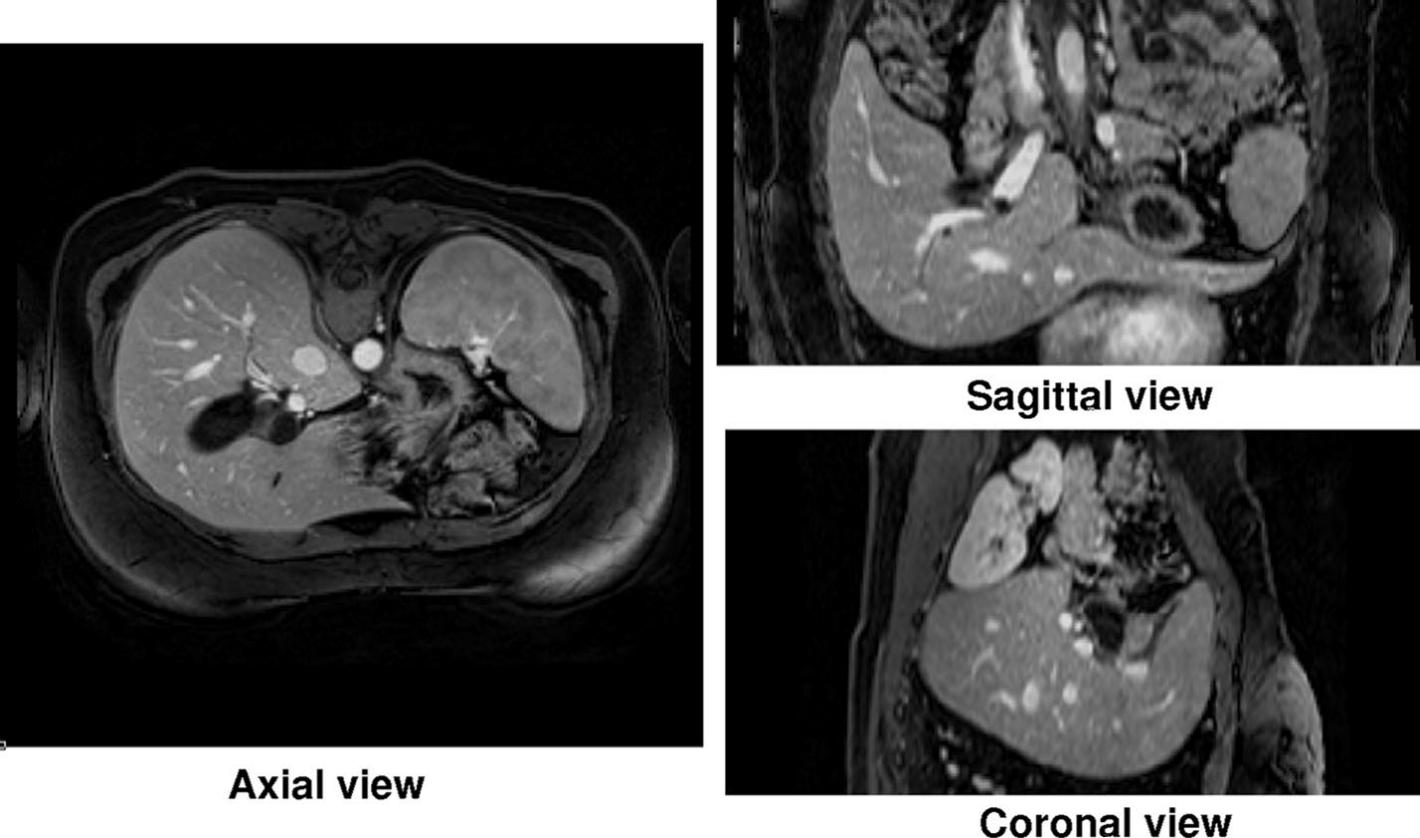
Example of original images, as captured by the MRI scanner

The statistical atlas was also built with C++ programs and functions from the ITK but requires further explanation. Since the method is based on landmark coordinates, a method to extract automatic landmarks from the binary shapes was needed. Furthermore, the number of landmarks in each case must be the same and they should be consistent, i.e.: orderly generated so that it can be assumed that the $i$-th mark of all cases corresponds to an anatomically equivalent point. Automatically generated landmarks (also known as semi-landmarks) are quite common [37]. Our idea to generate them consists of slicing the liver along each dimension (*x*,*y*,*z*) with appropriately separated slices. The separation is determined so that the number of slices along each dimension is the same for all cases (i.e.: it is normalized to take into account length/width/height of each shape). Then, for each slice the longest closed curve is found. Its length is normalized and points distributed along it are taken as the landmarks. The distribution is taken regularly along the curve length and the number of points, even different for each slice (it is made to depend on the average length of the intersection curves) is the same for the corresponding slice of all shapes. Principal component analysis is applied to the landmark coordinates and the result is also a set of landmarks with its corresponding variation modes. Since we need a binary shape to make significant comparisons, the inner space was filled using a viscous reconstruction procedure similar to the one described for 2D images in [38]. Viscous reconstruction is a morphological process that starts from a seed and makes steps of successive openings, dilations, and intersection with the marker image where in this case the marker image is precisely the set of landmarks. Viscous reconstruction tends to underestimate the shape (it is a smaller shape, contained inside the real one). To overcome this problem sometimes viscous reconstruction is applied twice: to the inner part of the shape, starting with an internal point as the seed and to the outer part, starting with an outer seed. The final result is the volume enclosed by the geodesic skeleton (in 3D, a surface) of the difference set between the outer and inner reconstructions. In our case this was approximately calculated as the Baddeley–Molchanov mean shape between the inner and outer reconstruction. These steps are shown in Fig. 1: automatically extracted landmarks, the viscous reconstruction starting with an inner seed, the inverted viscous reconstruction of the complement of the shape starting with an outer seed and the geometric mean of the inner and the outer reconstructions, which is taken as the result.

### Measures of goodness

The problem of evaluating the quality of an atlas, in our opinion, has not been widely addressed in the literature. The atlas is sometimes used to evaluate other things, commonly the quality of a segmentation like in [39, 40] or the accuracy of models of light propagation within the head [41] but the atlas itself is rarely, but occasionally [42] assessed. First, its final quality is related to the initial segmentation of the shapes used to build it and, even in manual segmentation procedures, with the protocols of organ or lesion delineation [43]. It is not clear which quality indexes should be taken and normally those used to evaluate the quality of the geometric coregistration are used for atlases, too. Some of these measures are widely accepted for evaluating binary shapes like Dice index, Jaccard index and Hausdorff distance. Among those used for comparing real-valued images, the mean of squared differences or the mutual information are common choices. When the atlas is a binary shape (or a set of them) , the Jaccard, Dice or Hausdorff are adequate to compare with a ground truth shape, and also other measures like compactness, specificity and generality as mentioned in [44]). But in the case of probabilistic atlases the value of the probability has to do mainly with the location of the point and therefore has no direct relationship with the signal value of a real case. The obvious example is a more or less homogeneous organ like a liver whose signal value in a MRI image is similar at all of its inner points and changes abruptly only near its outer surface but the probability of belonging is higher for deep points and decreases more smoothly near the border. This means that comparing probability with signal value may not be a good approach, even if we adjust or normalize it, and that other measures should be proposed to evaluate probabilistic atlases.

In this work we aim to exhaust all possibilities so when binary shape differences are evaluated, the most popular indexes will be used. In all cases it is assumed that both shapes are referred to a common frame and *V*(*S*) will stand for the volume of shape *S*.

- Dice or Sørensen-Dice coefficient:
  8 $$\begin{aligned} Dice(A,B)=\frac{2V(A\cap B)}{V(A)+V(B)}. \end{aligned}$$
- Jaccard index or Jaccard similarity coefficient:
  9 $$\begin{aligned} Jac(A,B)=\frac{V(A\cap B)}{V(A\cup B)}. \end{aligned}$$
- Hausdorff distance:
  10 $$\begin{aligned} H_d(A,B)=\max \left\{ \sup _{x\in A}\inf _{y\in B}d(x,y),\sup _{y\in B}\inf _{x\in A}d(x,y)\right\} \end{aligned}$$

Dice and Jaccard indexes are real numbers in the interval [0, 1]. Hausdorff distance is a real number greater than or equal to 0. Dice coefficient is not a distance between shapes (the triangular inequality does not hold) nor is it Jaccard, at least directly, but the quantity $d(A,B)=1-Jac(A,B)$ is.

Most of the literature in anatomical shape analysis uses the Dice coefficient, which is not always the best choice. Being based exclusively on volume, it does not take into account differences of shape corresponding to few, but very significant voxels, specially those belonging to thin, long structures (for instance, veins or a rib that incorrectly appears as part of a vertebral spine segmentation).

Evaluation of the probabilistic atlas is more difficult. The main interest in having such an atlas is to use it as an aid for segmentation [45]. In order to test its ability to fit well to a new shape not previously seen, evaluation must be done with respect to shapes not used to build the atlas. Moreover, it cannot be matched against original, unsegmented cases since, as previously argued, signal values are not indicative of probability and therefore it is not yet possible to see to what extent the atlas matches the organ. The approach we have taken is to use a leave-one-out procedure that builds the atlas using the several proposed approaches with all but one patient and then to compare the result with the correctly segmented organ (ground truth) of that patient; this is a common approach for this purpose, like in [46] or [17]. The comparison between the segmented (binary) ground truth and the probabilistic atlas (real-valued) is done in two ways: first, by defining as a figure of merit the average integral of the probability and second, by thresholding the atlas and using the previously mentioned measures between binary shapes.

We are working with random compact sets contained within a set *W*. Mean probability ($Int_p$) of a probabilistic atlas *P*(*x*) inside a shape *S* is defined as

11 $$\begin{aligned} Int_p(P,S)=\frac{1}{V(S)}\int _S p(x)dx - \frac{1}{V(W \setminus S)} \int _{W \setminus S} p(x) dx. \end{aligned}$$

The integral $\frac{1}{V(S)}\int _S p(x)dx$ is a sensibility measure. We measure the mean value of the probability over the region that should be considered within the set *S*. The value $1- \frac{1}{V(W \setminus S)} \int _{W \setminus S} p(x) dx$ is a specificity measure. The difference considered in equation 11 would be greater for a higher sensibility and specificity. This measure is in $[-1,1]$ and an atlas is more similar to a shape if $Int_p(P,S)$ is close to 1. In any case, lower values correspond with a worse performance. It is not a distance since it compares heterogeneous entities (binary shapes and probability maps) but intuitively. If all shapes used to build the atlas were exactly the same, the atlas would be a degenerate distribution taking 0 and 1 as its only values and identical to any of the shapes which would yield 1 as the value for $Int_p$. A case close to this happens when the geometric transformation used to build the atlas is too flexible and eliminates the original variability.

With respect to the second possibility, thresholding the atlas to use the classical measures between binary shapes, an important point is the choice of the threshold. In order to provide fair opportunities to all methods, a range of thresholds $(0,0.1,\ldots ,0.9)$ for *P*(*x*) has been tested and that which gives the best average result for each method has been chosen.

It is important to note that, when using powerful deformable methods, most authors use the same model of deformation, just inverting the transformation, to adapt the atlas to the case at hand. Results are obviously very good but this is only possible when the concrete case has already being correctly or almost correctly segmented. We emphasize that our results are useful in the opposite case: that in which the atlas is to be used to aid in the segmentation of a new case.

### Experiment condition and materials

The anatomical shapes used in this paper are binary shapes of manually segmented livers. The original images are dynamic perfusion magnetic resonance images (MRI) of the abdominal cavity. There were a total of 39 explorations from 21 different patients, 13 men and 8 women, with ages between 15 and 73. Anthropometric data is given in Table 1.

**Table 1:** Anthropometric data of the patients

Sex	Age	Weight (kg)	Height (m)
Female	42	56.3	1.58
Male	51	78.2	1.76
Male	59	75.2	1.72
Male	46	89.6	1.84
Female	68	49.5	1.52
Female	73	52.3	1.56
Male	32	74.5	1.75
Male	15	57.9	1.68
Female	28	65.1	1.74
Male	81	78.8	1.76
Male	79	81.2	1.72
Male	63	72.8	1.68
Male	58	70.7	1.76
Female	65	51.2	1.56
Male	51	72	1.70
Male	47	81	1.78
Female	48	68.5	1.72
Female	39	64.5	1.68
Male	67	90.3	1.88
Male	65	86.2	1.78
Female	42	70.3	1.66

**Fig. 2:**
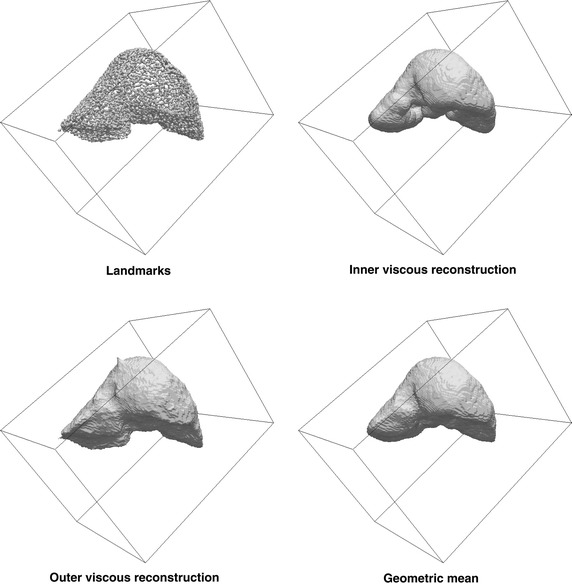
Statistical atlas reconstruction steps

All volumes were taken under the same conditions. The scanner was from Philips Medical Systems (model Ingenia 3.0T) and worked at 3T in Enhanced T1 High Resolution Isotropic Volume Excitation mode. Patients were in supine, head first, transverse orientation and they were required to not breathe as much as possible during the acquisition. Image resolution was of $256\times 256$ pixels per slice and 133 slices per volume being slices orthogonal to the machine displacement axis which was the axial (transverse) axis of the patient’s body. The real dimensions of each voxel were $1.46\times 1.46 \times 1.5$ mm, being 1.5 the separation between consecutive slices. For those patients with two or three explorations (17 of them), they correspond to different time instants of the contrast diffusion. A typical example with coronal, sagittal and axial sections of one case is shown in Fig.  2.

**Fig. 3:**
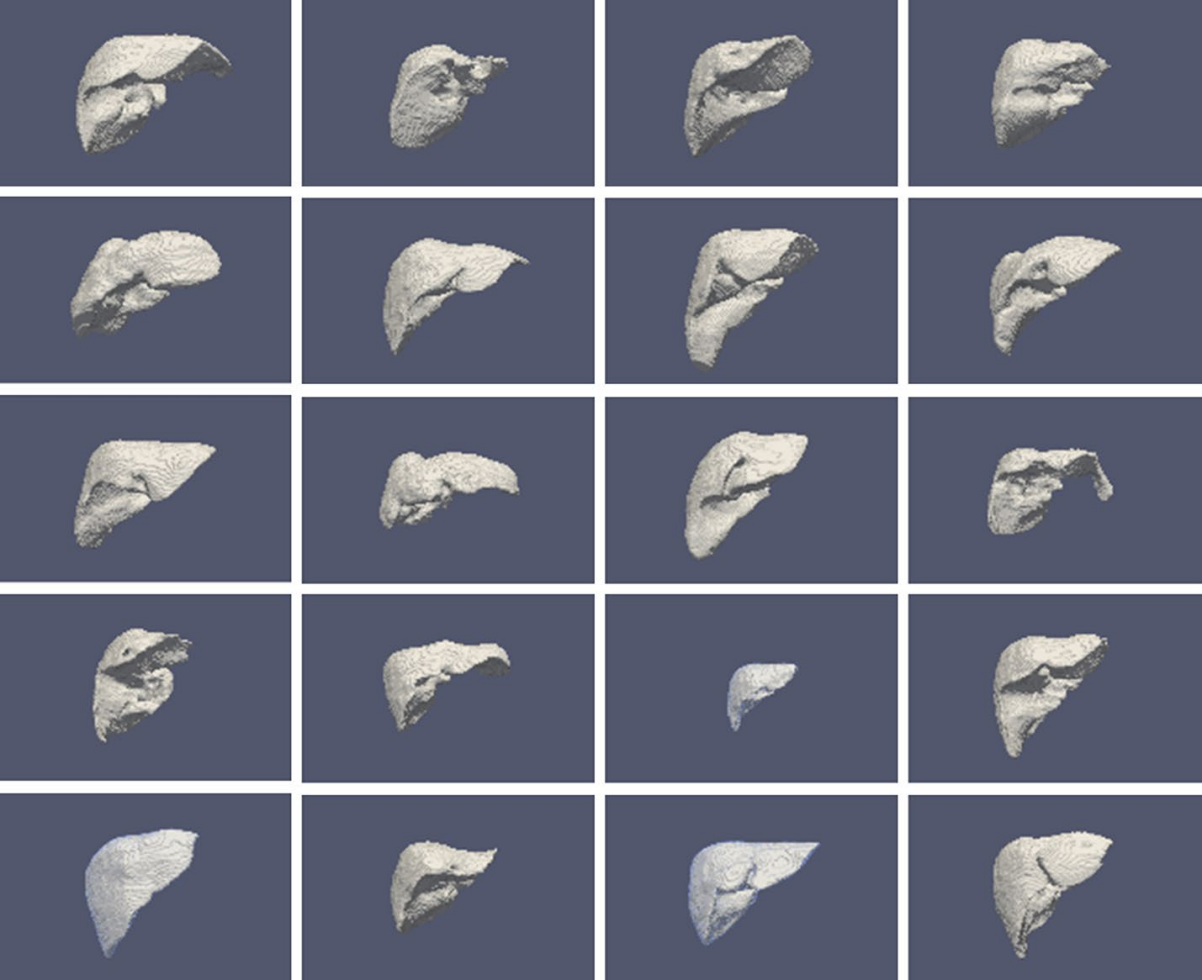
Mean shapes per patient of the segmented cases used in the study

The segmentation of the liver was done manually by an expert radiologist using software from the group and a pen-tablet type computer to delineate the contour in each slice and use that contour as the initial mark for the next slice, which can be deformed at any point using the pen. Even with such aids, the process is slow and may take up to two hours per exploration. As an illustration, the mean shape (using only the signed mean distance function thresholded at 0) of each patient, or the only shape for the cases of only one shape per patient, are shown in Fig. 3.

## Experimental study

### Graphical illustration

**Fig. 4:**
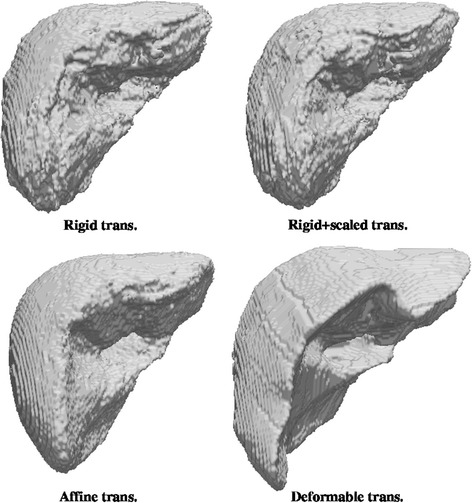
Binary shapes obtained thresholding the NCF atlas at 0.5 using the four coregistration methods

**Fig. 5:**
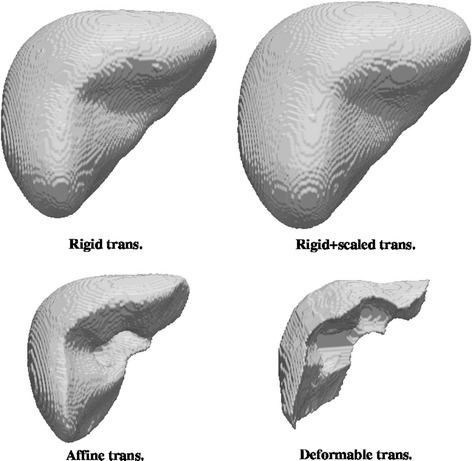
Binary shapes obtained thresholding the GLM atlas at 0.5 using the four coregistration methods

**Fig. 6:**
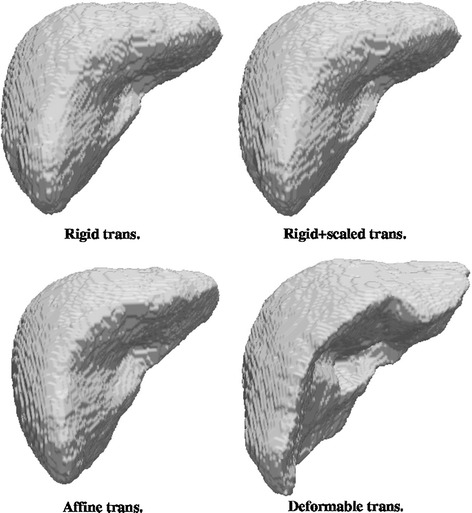
Statistical atlas using the four coregistration methods

**Fig. 7:**
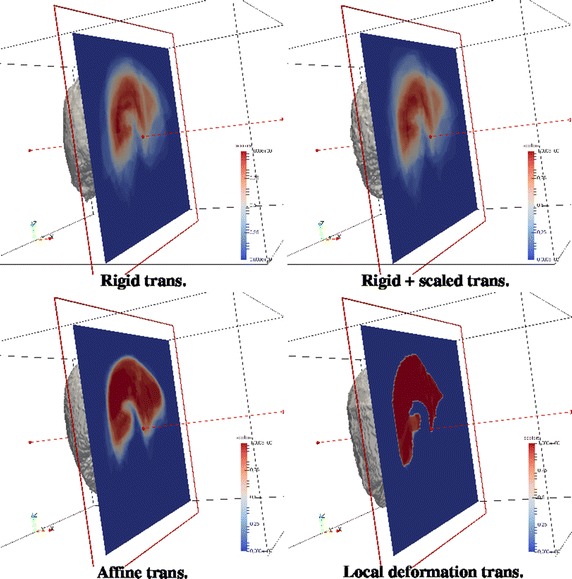
An axial cut of the probabilistic CF atlas. *Red* means higher value of probability, *blue* lower values

**Fig. 8:**
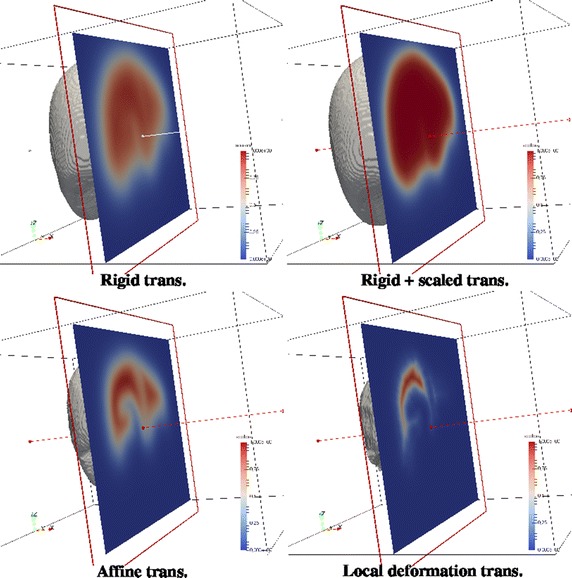
An axial cut of the probabilistic GLM atlas. *Red* means higher value of probability, *blue* lower values

From now on, abbreviations for methods will be NCF (normalized coverage function), GLM (generalized linear model) and STA (statistical atlas) followed by R (rigid transformation), TRS (translation and rotation plus global scaling), A (global affine transformation) or D (local deformable transformation). The following figures show the atlas obtained using all cases with the three proposed methods and with the four coregistration methods. For the NCF and GLM methods, whose result is a probability map, what the Figs. 4 and 5 show is the threshold at level 0.5 (a binary shape). Figure 6 shows the results of the statistical atlas method. Also, for the NCF and GLM, slice cuts with the probability scale are depicted in Figs. 7 and 8. All figures of the same type are shown with the same scale and seen from the same point of view.

Regarding the visual appearance of the binary shapes (Figs. 4, 5, 6), a comparison among the three atlas methods show that a rougher outer surface appears with the NCF; such surface is quite smooth for the statistical atlas and very smooth for the GLM. This is clearly a consequence of considering the global influence of all voxels through the linear model. Smoothness in this context (anatomical modelling) is usually considered a desirable property and indeed smoothness constraints for the limit surfaces are sometimes requested in segmentation algorithms. On the other hand, excessive uniformity may provoke the loss of fine details. In the case of the GLM this can be adjusted through the model parameters, which is not possible for the other methods. The size (volume) of the shapes is also a remarkable feature. The real size is only correctly reflected by the statistical approach since this method works with real coordinates whereas for NCF and GLM what it is shown is an arbitrary cut at some probability value (here, 0.5); but if size is a relevant consideration, other cuts of GLM or NCF can be used so that the enclosed volume approximates the mean volume of the shapes. Finally, the visual shape (to what extent the atlas resembles a real liver) is clearly dependant on the registration method: more complex geometric transformation give in general a better look, with the remarkable exception of the GLM with local deformation, which leaves out a great part of the liver showing what resembles artificial resections. But contrarily to intuition, and as it will be shown in “Numerical results” section, a less appealing shape like that given by the GLM with rigid plus global scaling transformation is nevertheless more effective in terms of appropriately reflecting the probability of belonging to the liver.

Figures 7 and 8 show part (left half) of the binary shapes already shown together with a plane cut depicting the probability given by the probabilistic atlases as color scale. Looking at the NCF method, the limits of the liver are better located as the complexity of the registration methods increases, up to to the point (local deformation) in which that atlas is almost completely made of only close to 0 and close to 1 probability values, resembling a binary shape. As stated before, this is not beneficial for keeping the variability of the sample of shapes. Looking now at the GLM method, the former comments are applicable, too, and furthermore the local deformation is particularly harmful for this method: a substantial amount of the inner part of the liver disappears (is considered background) because not enough shapes of the sample, after being registered to one of them, occupy that area. This might be corrected by choosing another shape as the registration basis but there is no objective criterion in advance. The shape that gave less than the sum of squared differences in registration with respect to all others (which was our choice) it is not always the best. In any case, not too flexible coregistration methods should be used for building an atlas with GLM since the linear model needs an amount of variability in the inputs to give account for new cases.

### Numerical results

The proposed experiment is a leave-one-out procedure which allows a sufficient sample size to get statistically sound conclusions and also makes sure the tests are done using an atlas with respect to a new case. First, all cases were coregistered using each of the geometrical transformations described in “Different models for registration” section.

Then, for each of the 39 explorations, atlases were built with all the explorations from the other patients using the three analyzed methods: STA, CF and GLM. Finally, each atlas is compared with the case left out using the measures stated in “Measures of goodness” section. Nevertheless, please notice that the STA atlas is only binary so the *IProb* measure defined in Eq. 11 cannot be calculated. Also, the Dice (Eq. 8), Jaccard (Eq. 9) and Hausdorff (Eq. 10) need binary shapes so their given values for probabilistic atlas methods (CF and GLM) are the optimal ones, i.e., the thresholded versions that gave the best result for each case. The values that will be shown in all tables of results are means of each measure applied to each of the leave-one-out atlas with respect to all other cases. The mean values together with their standard deviations are shown in Table 2.

**Table 2:** Mean values and standard deviations of each measure for each of the used registration and atlas building methods

	Hausdorff	Dice	Jaccard	IProb
NCFR	38.33 ± 15.49	0.74 ± 0.07	0.60 ± 0.09	0.63 ± 0.07
NCFTRS	38.49 ± 15.92	0.74 ± 0.06	0.60 ± 0.08	0.62 ± 0.06
NCFA	39.45 ± 16.62	0.73 ± 0.08	0.59 ± 0.10	0.67 ± 0.10
NCFD	39.24 ± 16.38	0.71 ± 0.09	0.56 ± 0.12	0.68 ± 0.10
GLMR	38.37 ± 13.71	0.73 ± 0.06	0.58 ± 0.08	0.69 ± 0.05
GLMTRS	38.07 ± 13.82	0.70 ± 0.07	0.55 ± 0.08	0.79 ± 0.03
GLMA	40.34 ± 15.97	0.73 ± 0.08	0.59 ± 0.10	0.57 ± 0.07
GLMD	42.92 ± 17.08	0.67 ± 0.07	0.50 ± 0.09	0.31 ± 0.03
STAR	43.87 ± 17.64	0.73 ± 0.06	0.58 ± 0.08	–
STATRS	44.09 ± 17.68	0.73 ± 0.06	0.58 ± 0.07	–
STAA	46.23 ± 18.38	0.72 ± 0.08	0.57 ± 0.09	–
STAD	43.62 ± 16.86	0.71 ± 0.09	0.56 ± 0.11	–

Once the mean values have been found, and considering separately each similarity measure, we can analyze differences between methods. Each possibility is compared against all the others by means of a t test with the null hypothesis of equality of means. The combination of the three atlas methods with the four registration methods gives a total of 12 possibilities; therefore, there are 66 possible paired tests between different possibilities. The threshold of adjusted p value for considering two methods as significantly different is, as usual, taken as 0.05. The results given in Tables  3, 4, 5 and 6 highlight the comparisons whose p value is lower than this threshold. Each square of this table has either two or three lines which are the mean value for the considered measure (method in the upper-row in the first line, method in the left-column in the second line) and also the p value for the t test of difference of means in the third line, when it is significant, and left blank when it is not. For each comparison, the winner method is highlighted with the gray level of either, the upper row or the left column, as appropriate.

**Table 3:**
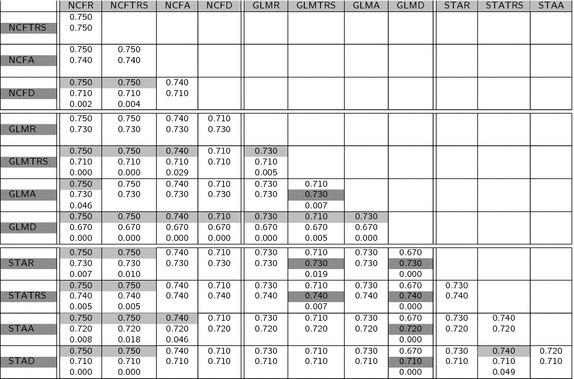
Mean values of Dice coefficient and p value for the paired test of equality of means

**Table 4:**
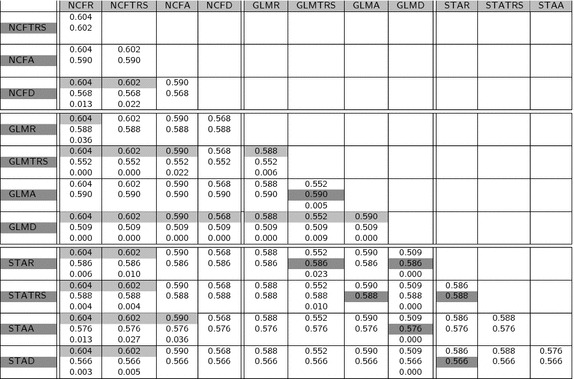
Mean values of Jaccard coefficient and p value for the paired test of equality of means

**Table 5:**
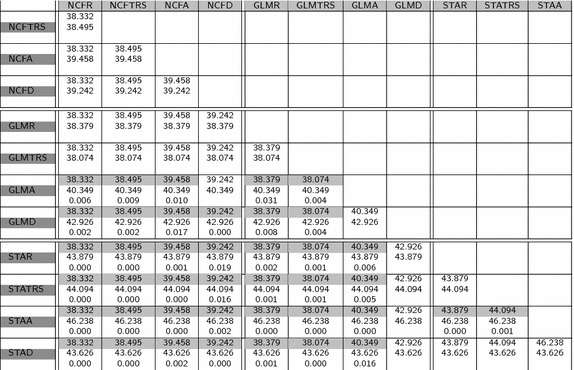
Mean values of Hausdorff distance and p value for the paired test of equality of means

**Table 6:**
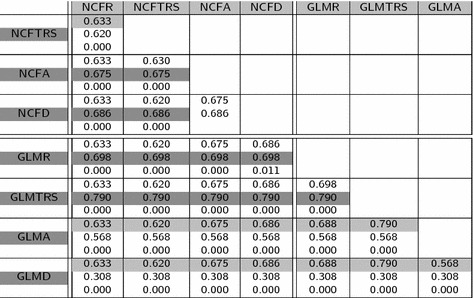
Mean values of IProb measure and p value for the paired test of equality of means

### Discussion

Regarding the table of mean values (Table 2), it is important to highlight that the Dice and Jaccard measures are quite similar for all the methods. This is also apparent by the fact that only about half of the paired tests provided in Tables 3 and 4 for Dice and Jaccard show significant differences between methods. Moreover, the significant differences do not follow a pattern related with the atlas method or the registration model. This suggests that the power of these measures to discriminate between different results is not optimal. We think this can be related to the fact of using only volume differences and not real shape differences to compare shapes. Hausdorff distance has also not too many significant differences. However, looking at the Hausdorff distance (Table 5) it can be observed that most of them are concentrated in tests comparing different atlas methods. This probably has to do with the ability of Hausdorff distance to capture shape differences even between shapes of similar volume or highly overlapped. The highest proportion of significant differences (all tests but one) correspond to the IProb measure (see Table  6). This assertion is reinforced, as well, by the observed variation of the mean values of each measure with respect to the global mean of it: Dice coefficient (second column of Table  2) is between 0.670 and 0.749, a variability of $11\%$ with respect to the mean. The same calculations for Jaccard coefficient gives 17, $18\%$ for Hausdorff distance and $45\%$ for the IProb. This leads us to concentrate for further analysis on the Hausdorff and IProb measures.

Focusing on Hausdorff distance between atlas methods, and concentrating on the coregistration, we can see that NCF does not show significant variations, whatever the registration method used (no NCFx to NCFy are significant). Similar results can be observed with STA with the exceptions of rigid and scaled rigid registrations being better than affine and local deformation. On the contrary, GLM is significantly different in four out of six comparisons, being again rigid and scaled rigid better than affine and local deformable registrations.

Comparing now different atlas methods, again in terms of Hausdorff distance, NCF outperforms STA for all registration models and also GLM for the affine and deformable models (but it is not significantly different for the others). Finally, GLM outperforms STA for all but deformable registration models and it is not significant in the deformable case.

The conclusions drawn from the Hausdorff distance are that NCF and GLM are quite similar, and better than STA and that the registration model should not be too complex. Although introduction of local deformations is visually appealing and highly precise for a particular case, it increases shape differences between atlas and new, unknown shapes.

Looking now at the IProb (Table 6), as stated before, all comparisons but one show significant differences. Separately for each atlas method, NCF is better when using affine or deformable models. We think this was not appreciated with Hausdorff distance because HD looks specially at the shape boundaries whereas IProb takes into account the whole volume values. GLM with itself is also different according to the registration model. Again, rigid and rigid plus global scaling are better than affine and deformable, and particularly rigid plus global scaling, which is also better than rigid only, gets a quite good performance (i.e.: the average probability of this atlas into a new shape is a high value, 0.79). Comparing now both atlas methods, GLM is better than NCF for the simple registration methods (rigid and rigid plus global scaling) but worse for the complex ones (affine and local deformable). The smoothness introduced by the linear model with respect to the raw data of the NCF has advantages as long as the registration method does not lessen the sample variability.

Finally, we must point out the limitations of this study. First, not every possible registration model has been used but only four of them because they were considered as representative of widely used choices. It is also true that the local deformation algorithm can in principle account for the most general deformation, there are several methods to estimate it and it is possible that more fine-tuned estimation methods could improve its performance. Also, the resulting atlas is only one and is applied to all shapes equally; other methods are based on the use of multiple atlases and the choice of one at the time of application. It might be possible, too, to apply different atlases to different regions of the image or at different scales. Despite all these limitations, we think this study still provides some valuable guidelines that can orient the choice of registration models and the methods to build an atlas, either if only one atlas is used or if a collection of them is to be generated.

## Conclusions and further work

The experimental study in this work was aimed to highlight the differences between atlas construction methods and its associated coregistration methods when used for anatomical purposes. The results show that, contrary to the first intuition, the use of a too complex registration method is not always the best choice. Keeping the variability is specially important, particularly for probabilistic atlases, if one wants the atlas be useful for segmentation of new cases. Among the tested methods we have proposed a new one for probabilistic atlas construction based on a sensible combination of probability of coverage and distance function by using a generalized linear model. Also, a new measure of performance of probabilistic atlases (IProb) that takes into account all the points of the volume has been proposed, showing its advantages in terms of discriminatory power with respect to more simple, frequently used measures based only on overlapping voxels. The combination of the proposed GLM atlas with a relatively simple registration model exhibits a good performance and seems a promising methodology. It remains to be proved to what extent these conclusions are valid for other organs or shapes which is left as future work; preliminary results are being carried out using vertebrae and spinal cord in [45].

## Abbreviations

PCA: principal component analysis
LogOdds: natural logarithm of the odds ratio
GLM: generalized linear model
MRI: magnetic resonance image
CT: computed tomography
NCF: normalized coverage function
R: rigid transformation
TRS: translation and rotation plus global scaling
A: global affine transformation
D: local deformable transformation
